# Insecticide susceptibility of *Anopheles* mosquitoes changes in response to variations in the larval environment

**DOI:** 10.1038/s41598-017-03918-z

**Published:** 2017-06-16

**Authors:** Henry F. Owusu, Nakul Chitnis, Pie Müller

**Affiliations:** 1 Epidemiology and Public Health Department, Swiss Tropical and Public Health Institute, Socinstrasse 57, PO Box, CH-4002 Basel, Switzerland; 2 University of Basel, Petersplatz 1, CH-2003 Basel, Switzerland; 3 Department of Immunology and Infectious Diseases, Harvard T.H. Chan School of Public Health, 665 Huntington Ave, Boston, MA 02115 USA

## Abstract

Insecticide resistance threatens the success achieved through vector control in reducing the burden of malaria. An understanding of insecticide resistance mechanisms would help to develop novel tools and strategies to restore the efficacy of insecticides. Although we have substantially improved our understanding of the genetic basis of insecticide resistance over the last decade, we still know little of how environmental variations influence the mosquito phenotype. Here, we measured how variations in larval rearing conditions change the insecticide susceptibility phenotype of adult *Anopheles* mosquitoes. *Anopheles gambiae* and *A. stephensi* larvae were bred under different combinations of temperature, population density and nutrition, and the emerging adults were exposed to permethrin. Mosquitoes bred under different conditions showed considerable changes in mortality rates and body weight, with nutrition being the major factor. Weight is a strong predictor of insecticide susceptibility and bigger mosquitoes are more likely to survive insecticide treatment. The changes can be substantial, such that the same mosquito colony may be considered fully susceptible or highly resistant when judged by World Health Organization discriminatory concentrations. The results shown here emphasise the importance of the environmental background in developing insecticide resistance phenotypes, and caution for the interpretation of data generated by insecticide susceptibility assays.

## Introduction

Insecticide resistance in mosquitoes, especially in malaria endemic regions, has increased dramatically in the last decade^1, 2^. With only four classes of insecticides available for adult mosquito control, and only pyrethroids accepted for long lasting insecticidalnets (LLINs), the World Health Organization (WHO) has scaled up efforts to control the rapid spread of resistance. These efforts were strengthened with the launch of the Global Plan for Insecticide Resistance Management in 2012^3^.

Attempts to reduce the likelihood of the development of resistance to insecticide as well as to improve the success rates of interventions against disease vectors have led to the promotion of an integrated approach to the control of vector-borne diseases^4^. These combined interventions have been shown to be effective in significantly reducing malaria morbidity and mortality^5^. Until the 1950s, larval source management (LSM) was the principal malaria control method^6^. The discovery of chemical insecticides saw LSM relegated to the background, but the recent increased preference for combined interventions has led to a resurgence of its application, and today, there is renewed interest in LSM to reduce mosquito populations^6^. There is strong evidence showing that eliminating breeding sites and targeting immature stages of mosquitoes significantly impact the incidence and prevalence rates of mosquito-borne diseases^7–10^. As a result, the WHO accordingly recommended larviciding as an important supplement to core interventions in 2012^11^. The growing interest in LSM also brings into the spotlight the interaction of the larval stage with the environment, since the implementation of LSM largely involves either manipulation or modification of the environment in which the larvae live^6^. All LSM activities aim at reducing larval population numbers, which directly or indirectly alter the level of competition for space and resources available to the surviving individuals.

The mosquito is dependent on the environment for the completion of its life cycle^8^, and the conditions encountered during the early developmental stages may have strong downstream effects on the life history traits. The larval stage is essential in the life cycle of the mosquito because it is the only immature stage where feeding takes place, making it important for the accumulation of nutritional reserves for the development of the adult in the pupal stage^12, 13^. The responses of the larval stage to varying conditions could therefore play an important role in population size regulation, and understanding these responses will contribute significantly to effective control measures. Mosquito life history traits such as larval survival, adult fecundity, longevity, susceptibility to viruses and size as well as biting behaviour have been shown to be affected by temperature, nutrition and population density during the larval stage in both field and laboratory populations^14–19^. At the centre of how the larva-environment-adult complex plays out is the role of body size in determining observed phenotypes. Changes in the larval stage can result in faster maturing offspring with larger body sizes^20, 21^ which have been argued to live longer, therefore increasing their potential to transmit diseases^22^. Adult body size, and for that matter weight, has been shown to influence several biological traits such as rates of gaseous exchange and metabolic rates^23^. The emerging interest in the importance of the larval stage has led to several studies investigating the impact of the environmental breeding conditions during the larval stage on different life history traits of the imago^24–27^. Little has been done, however, to understand and quantify its relevance to insecticide resistance. The complex nature of insecticide resistance requires a thorough knowledge and understanding of the environmental, physiological and genetic interactions that interplay between the larval and adult stages to produce observed phenotypes.

Currently, there are still a number of elementary questions that remain unanswered regarding which factors play a role in producing an observed phenotype in the susceptibility of mosquitoes to insecticides and the magnitude of their effects. The extent to which insecticide susceptibility test results are dependent on body size is also not well understood^28^. While previous studies have shown that larval nutrition and adult size correlate with susceptibility of the adults to insecticides^29, 30^, nutrition might not be the only factor involved and other elements may equally affect susceptibility or contribute to the effect of nutrition. The effect of breeding conditions on susceptibility may also vary between susceptible and resistance strains, due to differences in energy and resource usage and needs.

This study investigated the influence of temperature, nutrition and crowding during the larval stage and adult body weight on the susceptibility of *Anopheles gambiae* KISUMU1 and *A. stephensi* STI adults to insecticides. The implications of the observed effects for insecticide resistance monitoring are discussed.

## Results

Mosquito larvae were reared under two levels (high and low) each of temperature, nutrition and population density in a factorial experimental design. The emerging adults were tested against a previously established LC_50_ for permethrin in the WHO susceptibility assay. The estimated permethrin LC_50_ values for the STI and KISUMU1 colonies were 0.125% (95% confidence interval, CI: 0.023–0.255%) and 0.068% (95% CI: 0.018–0.118%), respectively. The observed 24 hour mortality against these LC_50_ values in the WHO assay varied between the treatment groups. In the STI strain, the highest mortality was recorded in treatment group “c” (low temperature, low nutrition and high larval density) at 79.7% and the lowest was 43.4% in group “b” (low temperature, high nutrition and low density). With 67.3%, group “ab” (high temperature, high nutrition and low density) had the lowest mortality in the susceptible KISUMU1 strain and group “ac” (high temperature, low nutrition and high density) had the highest mortality at 97.7%. A summary of the odds ratios (OR), p-values and 95% confidence intervals from the regression models in the two species is given in Table 1. From the models, nutrition had the biggest influence on mortality. Lower amounts of larval nutrition significantly increased adult mortality in both strains, with ORs of 4.4 (95% CI = 2.7–7.1, p < 0.001) in the KISUMU1 strain and 3.0 (95% CI = 2.2–4.1, p < 0.001) in STI. Larvae growing at lower levels of population density and temperature were found to be protective for the adults, in both the KISUMU1 (temperature: OR = 0.4, 95% CI = 0.2–0.7, p = 0.003; density: OR = 0.3, 95% CI = 0.1–0.5, p < 0.001) and the STI (temperature: OR = 0.7, 95% CI = 0.5–0.9, p = 0.02; density: OR = 0.5, 95% CI = 0.4–0.7, p < 0.001) models. While there was no interaction between any of the factors in the STI model, there was a significant interaction between temperature and population density in the KISUMU1 model (OR = 3.1, 95% CI = 1.3–7.3, p = 0.01). This indicates that the effect of temperature on mortality was different at low population density from the effect at high density in KISUMU1.

**Table 1:** The output of the regression model for the effect of the factors on mortality in the two colonies.

Colony	Factor	Odds ratio	95% CI	p value
STI	Nutrition (low)	3.0	2.2–4.1	<0.001
	Temperature (low)	0.7	0.5–0.9	0.02
	Density (low)	0.5	0.4–0.7	<0.001
KISUMU1	Nutrition (low)	4.4	2.7–7.1	<0.001
	Temperature (low)	0.4	0.2–0.8	0.005
	Density (low)	0.3	0.1–0.5	<0.001
	Interaction: Temp × Density	2.8	1.3–6.2	0.01

**Figure 1:**
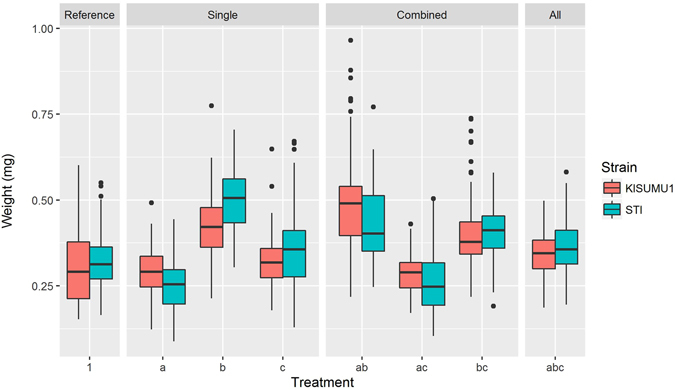
Weight distributions in the different experimental treatment groups. Treatments and treatment combinations are described in Table 4.

**Figure 2:**
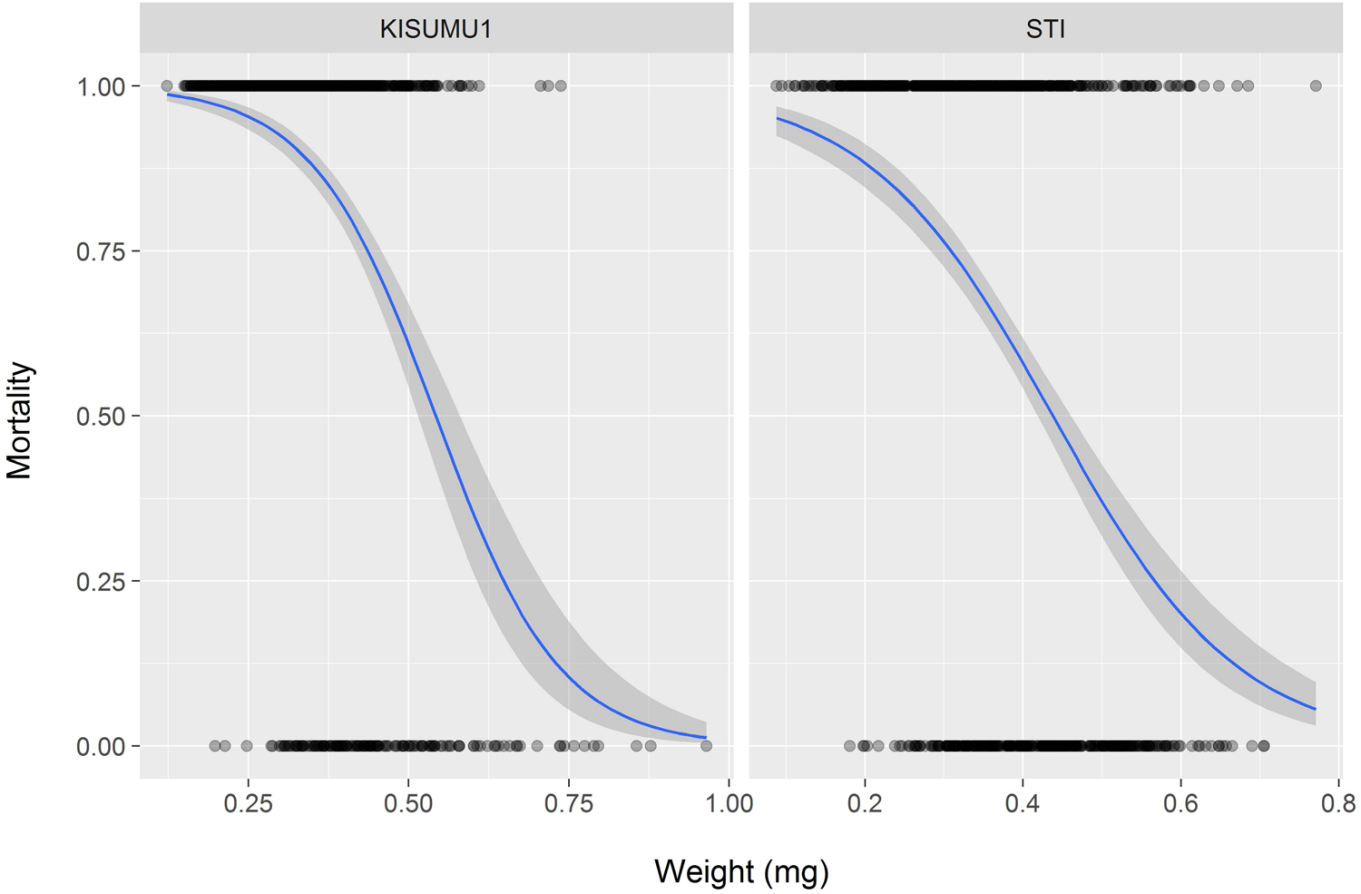
Mortality as a function of body weight. Dots represent individual mosquitoes (1 = dead, 0 = alive) and the lines show the predicted odds and the 95% confidence intervals (shaded areas) of dying as a function of weight.

After the WHO susceptibility assay, dead mosquitoes were separated from live ones and their dry weights were measured. The recorded weights varied considerably across the treatments in both colonies (Fig. 1) and ranged from 0.088 to 0.965 mg with a mean of 0.361 mg (95% CI = 0.356–0.368 mg). In both the STI (OR = 0.0002, 95% CI = 0.00004–0.0009, p < 0.001) and KISUMU1 (OR = 0.00003, 95% CI = 0.00004–0.0002, p < 0.001) strains, heavier mosquitoes were significantly more likely to survive the treatment with permethrin (Fig. 2).

Following the data obtained from the breeding experiment, nutrition turned out to be the most prominent factor affecting weight. We therefore adjusted the amount of food fed to the larvae to produce two new groups of mosquitoes (well fed and starved) with extreme body sizes to evaluate the practical implications of body size on susceptibility to insecticides. The “well fed” group was provided with 4x the amount given to the standard group per larva (Table 3) whereas the starved group was given ¼ of the standard amount. Emerging adults were tested in a dose-response assay and logistic regressions were used to estimate the LC_50_ values of the two groups.

**Figure 3:**
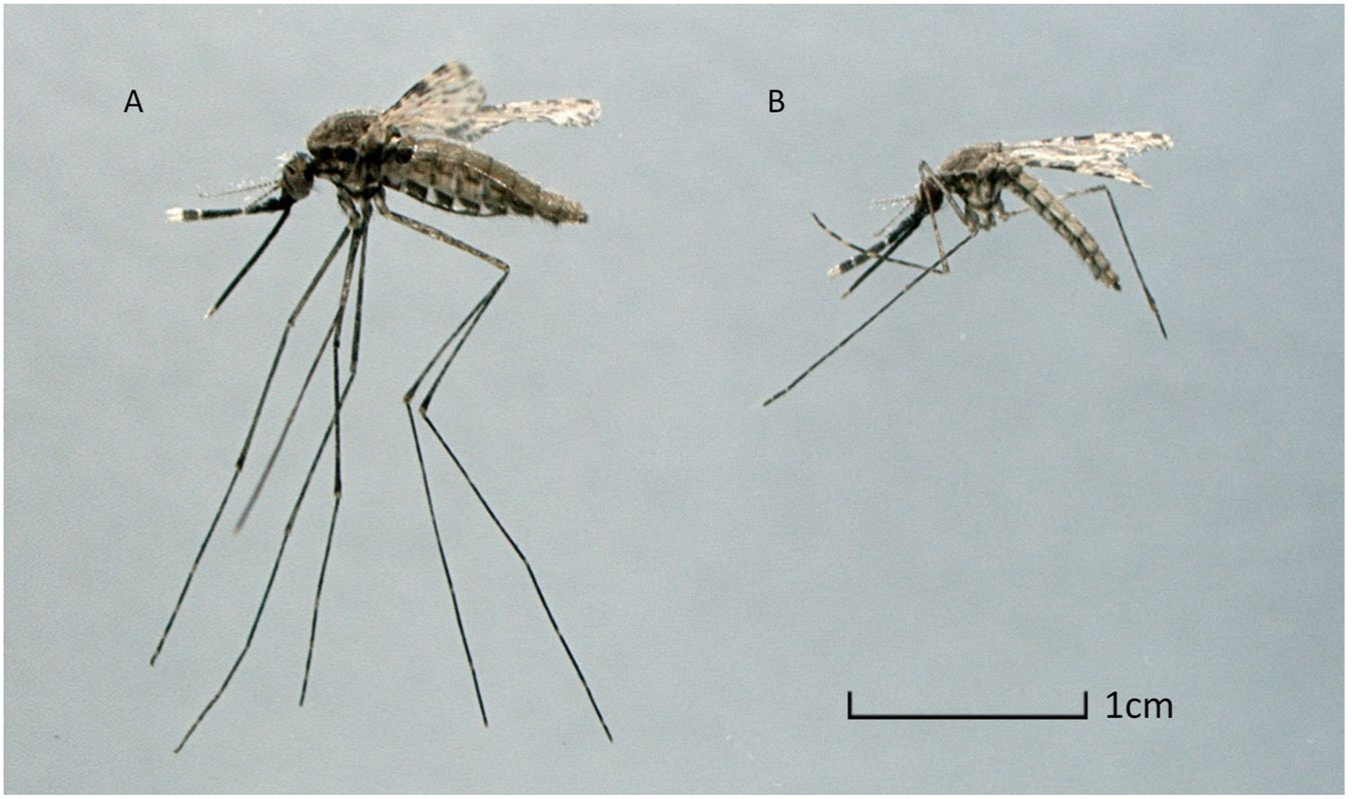
Examples of a well fed (**A**) and a starved (**B**) individual of the *A. stephensi* STI strain from the dose-response assay.

**Figure 4:**
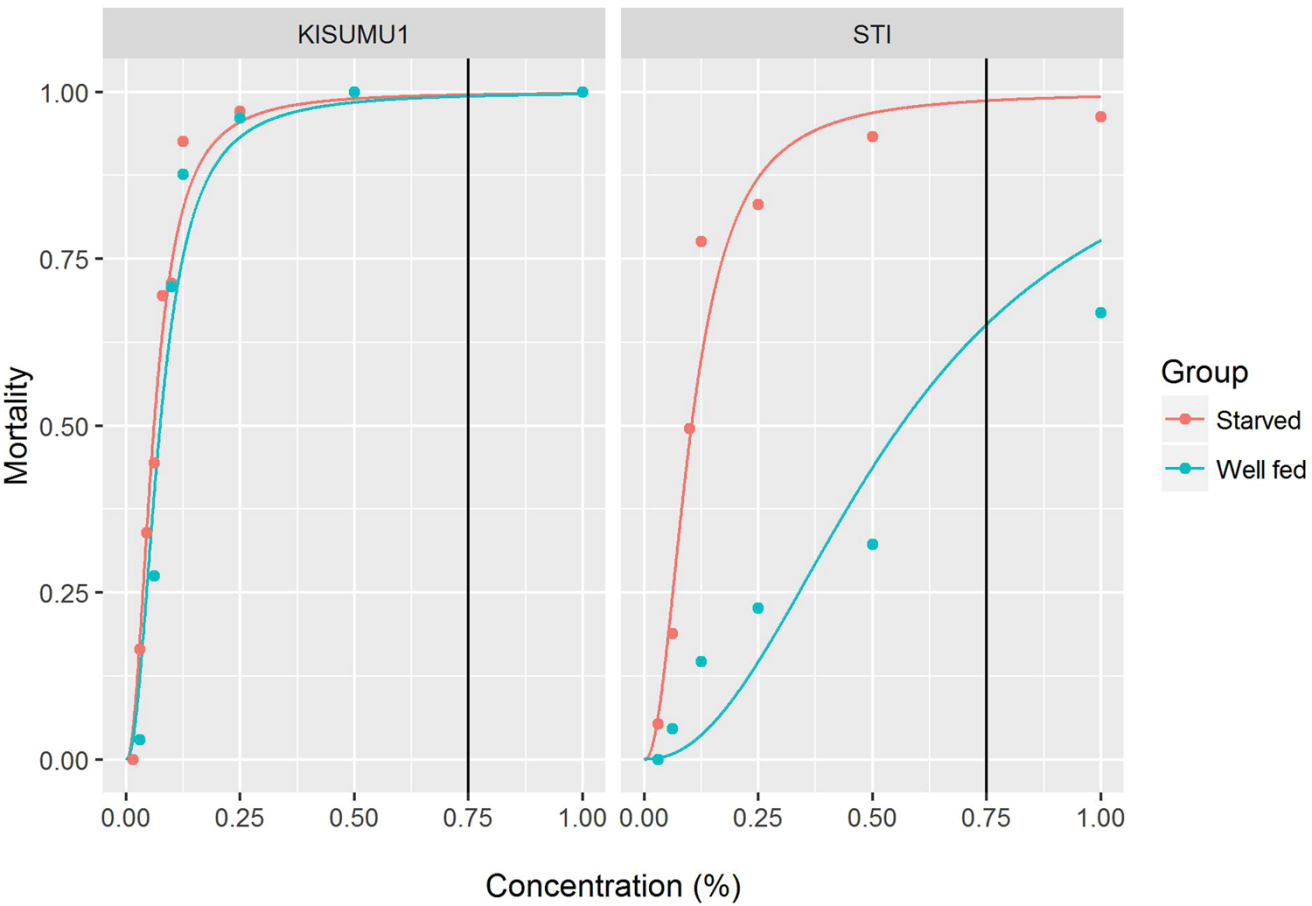
Dose-response curves for showing the mortality as a function of permethrin concentration for the starved and well fed groups. The dots show the summary mortalities measured at different insecticide concentrations on the filter papers in the WHO insecticide susceptibility assays. The lines are the predicted curves based on the statistical models.

In both strains, the well fed mosquitoes appeared larger than their starved counterparts as illustrated by the two specimens in Fig. 3. The predicted LC_50_ values (Table 2) showed that in both strains, the estimates for the two groups were higher in the well fed and lower in the starved groups than the values obtained in the reference groups (0.068% for KISUMU1 and 0.125% for STI). In KISUMU1, the predicted values for the well fed and starved groups were 0.079% and 0.062% respectively. There was a bigger shift in LC_50_ in the resistant STI strain in favour of the larger mosquitoes. The well fed group had an LC_50_ of 0.67% as compared to 0.103% for the starved group, a 6.5 fold increase. From the dose-response curve, the predicted mortality at 0.75% permethrin, the WHOPES discriminatory concentration^28^, showed a change in mortality between the groups in the STI strain but not in the KISUMU1 strain (Table 2, Fig. 4). The change is so dramatic that when starved, the population would be considered susceptible (i.e. 98% mortality), while in the presence of abundant nutrition the colony becomes resistant (i.e. 65% mortality) to permethrin.

**Table 2:** Predicted values of LC_50_ and mortality at 0.75% permethrin concentration from the two treatment groups compared against the observed mortality in the reference group.

Strain	Group	Nutritional amount^1^	LC_50_ [%] (95% CI)	Mortality at 0.75% permethrin (95% CI)
KISUMU1	Starved	0.25	0.062 (0.03, 0.121)	0.996 (0.994–1.0)
	Standard	1.00	0.068 (0.018, 0.118)	0.99 (0.941–1.0)
	Well fed	4.00	0.079 (0.011, 0.148)	0.998 (0.997–1.0)
STI	Starved	0.25	0.103 (0.005, 0.201)	0.98 (0.97–0.99)
	Standard	1.00	0.125 (0.023, 0.255)	0.71 (0.62–0.79)
	Well fed	4.00	0.670 (0.486, 0.854)	0.65 (0.59–0.71)

The figures for the standard group were observed values obtained from WHO insecticide susceptibility bioassays.

^1^The nutritional amount is given as the ratio of food provided as compared to the standard condition in Table 3.

## Discussion

The data presented here show that larval breeding conditions in *Anopheles* mosquitoes considerably influence the susceptibility of the adults to permethrin, and that adult dry weight is a very strong predictor of 24 hours mortality.

The effects of changes in larval breeding conditions on the fitness of the adult mosquito has been the subject of multiple studies. Both field and laboratory based studies, have shown that changes in environmental conditions during the larval stage significantly affect several life history traits such as larval survival^31^ and adult longevity^18^. The current study demonstrates that temperature, crowding and availability of nutrition during the larval stages directly or indirectly modulate 24 hours mortality in the adult when exposed to permethrin. There is strong evidence that decreasing larval density and higher nutrition lead to improved survival and faster development time in the immature stages as well as increased biting frequencies, longer survival, wing length and blood meal duration leading to increased vectorial capacity in the adult^13, 15, 32–34^. In relation to insecticide resistance, the current data are consistent with the findings of two previous studies in which *Anopheles* mosquitoes were exposed to DDT after varying larval nutrition^29, 30^. It was noted that nutrition might be the most important factor and that the effect of density could be compensated by the availability of food. From the data presented here, larvae that were bred under high density with high nutrition had lower mortality than those at low density and low nutrition.

In the current study, as an intermediate outcome, dry weight had a very strong association with mortality in both the insecticide resistant and the susceptible strains. Like in previous studies, at high population density, emerging adults were smaller^25, 35^ and more susceptible to insecticides. The effect of crowding could be the result of physical interactions rather than chemical, as suggested by Roberts & Kokkinn^36^ who observed that moving larvae constantly bumping into others and causing waves of disturbances in the feeding larvae, are the main cause of the crowding effect. Consistent with previous findings^37, 38^, the heaviest groups in both species were observed when nutrition was high and larval density was low. Just as in some preceding studies^16, 39, 40^, mosquitoes bred at the lower temperature weighed heavier than those reared at the higher temperature, although some studies found no association^41^. In his review, Atkinson found that about 90% of aquatic organisms showed a reduced body size at a higher temperature, and noted that this seemed paradoxical as higher temperatures would be expected to increase metabolism^42^. Possible explanations could be that organisms in higher temperatures have a higher metabolic rate and are also more active, thereby expending more energy, which results in the smaller body sizes or 32 °C is rather in the zone where larval development is on the decline and closer to the upper lethal temperature^43^. Although this study was conducted in the laboratory, the levels of the conditions experimented are not far off from field conditions. Larval breeding sites vary in size and it will not be unusual to have large numbers of larvae crowded in a small habitat or a few with abundant food. The temperature values chosen have also been recorded in field mosquito habitats^44, 45^.

As the focus on the effects of the environment on mosquito dynamics intensifies, the role of body weight in these dynamics is also becoming more apparent and it may hold an important key in future mosquito control strategies. It may be the most important physiological proxy in the observed interactions between the environment and the life history traits, since the majority of the beneficial traits have been observed in bigger mosquitoes. In addition to larger offspring being more tolerant to insecticides as demonstrated here, they have also been shown to be more efficient vectors by being better at finding hosts^34^ and having as much as a three-fold relative increase in transmission potential than smaller mosquitoes^32^. Although larger males might not be the most successful in finding partners during mating^46^, their female counterparts survive better and have a higher probability of being inseminated and producing more egg batches than smaller ones^22^.

The role of weight in defining mortality after treatment with insecticide might be linked to the amount of nutritional reserves available. Both wing length and weight are manifestations of body size^38^. But for the purpose of this study, dry weight was measured. This is because mosquitoes with similar wing size could still differ in weight even when bred under the same conditions due to their differences in the accumulation of proteins and lipids^47^. Studies have shown that the energy reserves in mosquitoes are size-dependent^13^ and larvae bred on high nutrition have higher reserves than those on a lower diet^48^. In other studies, resistant mosquitoes were found to contain less energy reserves than susceptible ones^49, 50^, suggesting that they use up the energy reserves to activate processes that overcome the toxins.

As much as dry weight is strongly associated with mortality, it is an interesting observation that there is no such association between weight and time-to-knockdown (Supplementary Information online). This emphasises that time-to-knockdown and mortality are not directly linked^51^ but rather more complex and the two endpoints do not share the same predictors.

A shortfall of this study is that changes at the genetic level were not measured. Lehmann^38^ indicated that there is substantial genetic contribution to phenotypic variations observed in adaptive traits. It would therefore be valuable to know how the phenotypic changes observed are genetically controlled. It would also be important to find out what kind of resistance mechanism is acquired, if any. As mentioned, it has been shown before that changes during the larval stage influences the insecticide susceptibility of the adult mosquito^29^, yet, this study went a step further by looking at both single and combined factors and as well as providing an insight into the practical implications of the effects by showing that in a resistant strain, LC_50_ can increase by about 6 fold when food is abundant.

The effect of incomplete larviciding on the population dynamics of mosquitoes is a very important subject for discussion; the fate of larvae that survive control activities will influence the overall impact of larviciding on malaria transmission, since the surviving larvae will develop under conditions of low density and high nutrition. From this and other laboratory studies^29, 30^ larval conditions affect insecticide resistance and the practical impact on vector control cannot be ignored. While resulting larger mosquitoes have several advantages, this study shows that the resistance threshold is significantly shifted in their favour as well. The predicted mortalities at the WHO discriminating concentration for permethrin (i.e. 0.75%) suggest that a change in weight has little effect in the apparent absence of insecticide resistance mechanisms but does have a considerable impact in the presence of resistance mechanisms. A further step in understanding the dynamics would be to evaluate field populations. Energetic reserves have been found to vary in different directions between field and laboratory populations^52, 53^ but a similar impact of weight on resistance as we found in this study may be expected.

The results of this study have considerable consequences for product development involving screening of insecticide resistance in lab colonies. Different breeding conditions may result in divergent adult phenotypes which may contribute to inter- and even intra-laboratory inconsistencies in bioassay results upon which vector control product efficacy assessments are based. Laboratories must be charged with maintaining high consistency in the density and amount of nutrition provided to larvae and regularly control weight or size of adults to ensure consistent test mosquitoes. It is, therefore, highly recommended that such practices are included in test guidelines and quality control procedures.

The findings from this study strengthen the importance of environmental contributions to the expression of resistance to insecticides in mosquitoes. The need to give environmental factors more attention in addition to the genetic background of mosquitoes, particularly in using resistant strains is strongly advocated.

## Methods

### Mosquito colonies and standard rearing conditions

Laboratory colonies of a pyrethroid resistant *A. stephensi* (STI) and a pyrethroid susceptible *A. gambiae* s.s. (KISUMU1, MRA-762) strains^54^ were used. The STI colony was obtained from the London School of Hygiene and Tropical Medicine in 1971 and the KISUMU1 strain was obtained from the Malaria Research and Reference Reagent Resource Center (MR4) in 2011. Adult mosquitoes were maintained in plastic 30 cm × 30 cm × 30 cm BugDorm cages (MegaView Science, Taiwan) on 10% sucrose solution at a temperature of 26–28 °C and a relative humidity of 60–74% in a 12:12 hours day:night regime. The females were fed with pig blood and 72 hours later, each cage was provided with an 80 mm diameter crystallizing dish, filled to a depth of about two centimetres with water to collect the eggs. To prevent the eggs from sticking to the glass and drying out, the rim of the dishes were lined with filter paper. The egg dishes were left in the cages for 24 hours after which they were removed and the harvested eggs were left in the dishes to hatch. Three hundred newly hatched larvae were then transferred using a Pasteur pipette into 30 cm × 19 cm × 8 cm larval trays filled to a depth of 1 cm with tap water treated with AquaSafe (Tetra, Germany). The larvae were fed on ground Tetramin fish food (Tetra, Germany) according to the protocol in Table 3.

**Table 3:** Feeding protocol used under standard rearing conditions at 27 °C and a density of 300 larvae per tray.

Day	Larval stage	Amount of food per larva (mg)
1	Hatching L1	0.1
2	L1/L2	No feeding
3	L2	0.1
4	L2/L3	No feeding
5	L3	0.4
6	L3/L4	No feeding
7	L4	0.4
8	L4/pupae	No feeding

In the case of extended development time, the larvae were fed 0.4 mg on alternate days.

### Larval rearing experiments

In a preliminary experiment, dose-response curves for the KISUMU1 and STI strains were estimated to define the lethal concentration at which 50% of the mosquito population would be killed (LC_50_) using the WHO susceptibility assay kit^28^. Three to five-day-old females obtained from larvae reared under the standard rearing conditions (see above) where exposed in WHO tubes to filter papers (Whatman No. 1) impregnated with permethrin (25:75 cis:trans ratio) at concentrations of 1%, 0.5%, 0.25%, 0.125%, 0.0625%, 0.0313% and a negative control containing no insecticide for 1 hour. The filter papers were prepared from mixtures of insecticides dissolved in acetone and Dow Corning 556 Silicon fluid. At each concentration, a minimum of 100 female mosquitoes were exposed in batches of 24 to 33 individuals. After the exposure, mosquitoes were transferred from the test to the holding tube, provided with 10% sucrose solution and held for 24 hours after which mortality was recorded.

In the next experiment mosquito larvae were reared under different rearing conditions and the emerging adults were exposed to the established LC_50_. The LC_50_ guaranteed that the effect of a rearing condition on the insecticide susceptibility could be measured in both directions, that is, either an increase or a decrease in permethrin susceptibility. Adult mosquitoes were reared as described above, and newly hatched larvae were split into different larval trays and each tray subjected to a specific rearing condition (i.e. treatment) to investigating the effects of temperature (A), population density (B) and the amount of food per larva (C) as well as their combinations. Each of the three factors was set at two levels, low and high, yielding 8 different combinations of rearing conditions (Table 4). Temperature was set either at 24 °C or 32 °C. Population density had a low level of 150 larvae per tray (half the standard conditions) and a high level of 600 larvae per tray (twice the standard conditions). Nutrition was either half or double the standard amount of food indicated in Table 3. The amount of food was adjusted for the larval density in order to separate the effects of nutrition from that of population density. The trays were then placed in a thermostatic cabinet (AQUALYTIC, Germany; Model AL654) to regulate air temperature, while the water level was checked daily and topped up when necessary. Temperature was continuously monitored with a Log 32 TH Data Logger (Dostmann Electronic, Germany). Emerging adult females were split by treatment and transferred to separate 30 cm × 30 cm × 30 cm plastic cages. Three to five-day-old adult females were exposed in batches of 20 to 28 to filter papers treated with the previously established LC_50_ in the WHO susceptibility test. After an exposure period of 1 hour, the mosquitoes were transferred into a holding tube, provided with 10% sucrose solution and held for 24 hours after which mortality was scored. Each treatment combination was replicated in at least 4 trays and a minimum of 100 adult mosquitoes were tested for insecticide susceptibility.

**Table 4:** Larval rearing conditions used in the factorial experiment.

Treatment	Rearing condition	Temperature (°C)	Food/density ratio^1^	Larvae per tray
1	Reference	24	0.5	150
a	High temperature	32	0.5	150
b	High food	24	2.0	150
c	High density	24	0.5	600
ab	High temperature, High food	32	2.0	150
ac	High temperature High density	32	0.5	600
bc	High food, High density	24	2.0	600
abc	High temperature, High food High density	32	2.0	600

^1^The food ratio indicates the ratio of the amount of food fed to the larvae as compared to the standard rearing conditions (Table 3). In the reference treatment all factors were set at the lower levels.

Based on the outcome of the larval rearing experiment, a second rearing experiment was carried out in which larvae from each colony were reared under the conditions that yielded the least and the most susceptible adults in order to assess the practical implications rearing conditions would have on the dose-response and the interpretation of WHO insecticide susceptibility assay data at the discriminatory concentration. Again a minimum of 100 females in batches of 22 to 32 individuals were exposed for 1 hour at the permethrin concentrations used to establish the initial dose-response curve. After the exposure, mosquitoes were transferred from the test to the holding tube, provided with 10% sucrose solution and held for 24 hours after which mortality was recorded.

### Measuring relationship between mortality and body weight

In order to measure the relationship between mortality and weight, dead and alive mosquitoes from the susceptibility assays were separated and their dry body weight measured. In preparation for the weighing, mosquitoes were transferred to punctured 1.5 ml Eppendorf tubes. While dead mosquitoes were transferred directly, live mosquitoes were first killed in a −20 °C freezer. The tubes were then left over silica gel in a sealed Tupperware box for at least 14 days to ensure complete and uniform drying. To avoid irregularities in dry weight introduced by re-absorption of atmospheric moisture, the weighing was carried out in a temperature and humidity-controlled cabinet. The mosquitoes were moved into the cabinet containing the balance at least 24 hours prior to weighing to condition them to the pre-set temperature and relative humidity. The temperature and relative humidity ranged between 21–25 °C and 30–40%, respectively. Once conditioned the mosquitoes were weighed to the µg using a UMX2 micro balance (Mettler Toledo, Switzerland).

To further understand the relationship between weight and mortality, we tested mosquitoes to investigate whether dry weight predicts time-to-knockdown in a modified CDC bottle assay^51^. The findings are provided as Supplementary Information which is available online.

### Data analysis

All data analysis was done in the freely available statistical software package R, version 3.2.0^55^. The LC_50_ values for the susceptibility assays were calculated on the basis of a predicted dose-response curve which was fitted assuming a binomial distribution and a logit link function. The effects of temperature, nutrition and density on mortality were analysed by generalised linear mixed-effect models (GLMM)^56^. In the GLMM, mortality was predicted by the independent variables temperature, population density and nutrition which were treated as fixed terms. Each treatment was replicated at least four times and the tray was introduced into the model as a random term to account for variability among trays. The GLMMs were modelled using the lme4 package in R^56, 57^. The relationship between mortality and weight was predicted by a logistic regression. The practical implications of different larval breeding conditions on the susceptibility of adults to insecticides were modelled by dose-response curves in logistic regressions which assumed a quasibinomial distribution. The models were run separately for the two species. The significance level was set at *α* = 0.05. All graphs were generated with the package ggplot2^58^.

## Electronic supplementary material

Supplementary Information
