# Supplementary material for: Insecticide susceptibility of Anopheles mosquitoes changes in response to variations in the larval environment
Source: Sci Rep. 2017 Jun 16;7:3667. doi: 10.1038/s41598-017-03918-z (PMC5473885; doi:10.1038/s41598-017-03918-z)
Supplement: Supplementary file 1 — Supplementary Information [file 41598_2017_3918_MOESM1_ESM.pdf]

Insecticide susceptibility of *Anopheles* mosquitoes changes in response to variations in the larval environment

Henry F Owusu[1, 2, 3], Nakul Chitnis[1, 2] and Pie Müller[1, 2]*

Supplementary experiment

Results

To further understand the relationship between weight and mortality and how it affects bioassay outcomes, we tested mosquitoes to investigate whether dry weight predicts time-to-knockdown in a modified Centers for Disease Control and Prevention (CDC) bottle assay. The choice of assay was to ensure that the mosquito is guaranteed to be in contact with the insecticide throughout the exposure time (which may not always be the case in the WHO susceptibility assay) and also for the ease of visualizing knockdown when it occurred. 76 female *A. stephensi* mosquitoes tested individually against permethrin showed no statistically significant association between dry weight and time-to-knockdown (coeff -3.16, 95% CI = -6.80 - 0.49, p = 0.09). But in agreement with the findings in the data from the breeding experiment, a logistic regression showed that heavier mosquitoes were more likely to be alive 24 hours after exposure (OR = 0.0001, 95% CI = 0.000001 - 0.013, p < 0.001). Supplementary Fig. S1 illustrates a scatter plot of time-to-knockdown against weight.

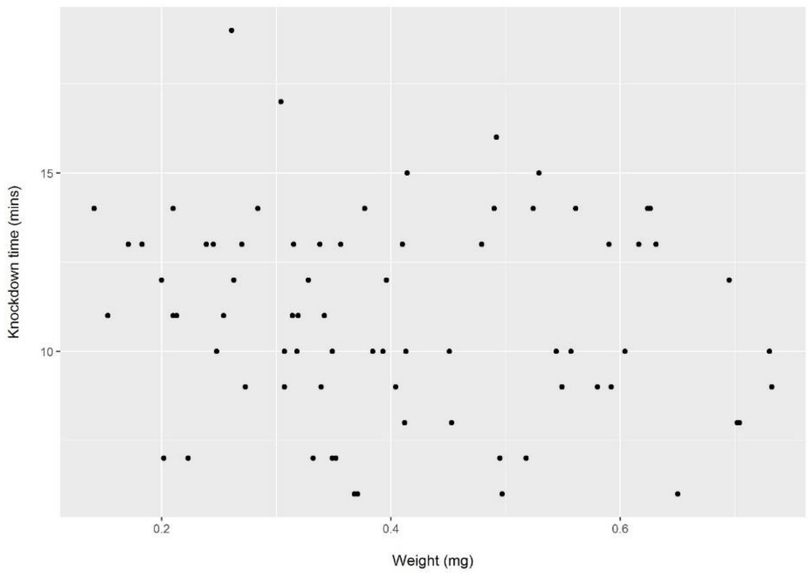

Supplementary Figure S1: A scatter plot of dry weight against time-to-knockdown in the STI strain against the CDC recommended diagnostic dose of permethrin

Method

The evening before testing, 250 ml SIMAX bottles (Kavalierglass Co. Ltd., Czech Republic) were coated with the CDC recommended concentration of 21.5µg permethrin (25:75 cis:trans ratio) dissolved in acetone. Three to five-day-old non blood-fed female *A. stephensi* mosquitoes bred under standard conditions were exposed to the treated bottles and observed for knockdown up to a maximum of 2 hours. Mosquitoes were exposed individually till they were knocked down so that both time-to-knockdown and 24 hours mortality of each could be easily tracked and compared. Once knockdown occurred, the mosquito was immediately transferred into a small plastic 30 ml beaker, covered with a small piece of cotton mosquito net and labelled with a unique identification number. The mosquito's id and time-to-knockdown were recorded. In addition, it was provided with 10% sugar solution and held for 24 hours after which mortality was recorded. Each mosquito was then dried over silica gel and weighed as described previously.
